# Academic freedom and innovation

**DOI:** 10.1371/journal.pone.0304560

**Published:** 2024-06-11

**Authors:** David B. Audretsch, Christian Fisch, Chiara Franzoni, Paul P. Momtaz, Silvio Vismara

**Affiliations:** 1 O’Neill School of Public & Environmental Affairs, Indiana University, Bloomington, IN, United States of America; 2 Interdisciplinary Centre for Security, Reliability and Trust (SnT), University of Luxembourg, Luxembourg, Luxembourg; 3 School of Management, Polytechnic University of Milan, Milan, Italy; 4 TUM School of Management, Technical University of Munich, München, Germany; 5 Department of Management, University of Bergamo, Bergamo, Italy; Maynooth University, IRELAND

## Abstract

Academic freedom is a critical norm of science. Despite the widely postulated importance of academic freedom, the literature attests to a dearth of research on the topic. Specifically, we know little about how academic freedom relates to indicators of societal progress, such as innovation. We address this research gap by empirically assessing the impact of academic freedom on the quantity (patent applications) and quality (patent citations) of innovation output using a comprehensive sample of 157 countries over the 1900–2015 period. We find that improving academic freedom by one standard deviation increases patent applications by 41% and forward citations by 29%. The results are robust across a range of different specifications. Our findings constitute an alarming plea to policymakers: global academic freedom has declined over the past decade for the first time in the last century and our estimates suggest that this decline poses a substantial threat to the innovation output of countries in terms of both quantity and quality.

## 1. Introduction

Freedom is recognized as a source of growth since the beginning of modern economic thought [1]. Free-market societies develop faster because freedom spurs innovation [2]. Institutions that allow free cooperation and competition promote the production and exploitation of knowledge [3, 4]. Independence from authority and hierarchy fosters an atmosphere of information exchange and tolerance to failures that spurs idea circulation, experimentation, diversity, and creativity, all ultimately inducing innovation [5, 6].

Similarly, academic freedom is embedded in the norms of science, along with other cornerstones of science, such as open disclosure and freedom of critique [7, 8]. These norms are key to promoting the kind of unconditional exploration that fuels science and would not be possible in the private sector, because of highly uncertain economic returns and limited appropriability discourage profit-oriented researchers [9–11].

Yet, universities are increasingly pushed to seek outside funding for their research and are incentivized to make an impact in the society. The consequences of this “third mission” on the level of research freedom and the ethical challenges of this increased reliance on outside funding are considered [12–14]. The fundamental question of whether the transformation of the university into a “research institution for the industry” could have negative effects on the research activity was already raised by [15]. While universities should abide by general ethical principles of creating public goods in terms of contribution to knowledge and betterment of all citizens, the industry’s fundamental logic is to pursue profit. With some exceptions [13], this important question has not sparked a general discussion on the ethical implications of sponsored research more than three decades later.

Reopening the debate on academic freedom and its link to innovation seems especially important today, with indicators registering signs of a decline in academic freedom over the last decade for the first time since World War II [16]. This sentiment is picked up by policymakers, with the European Parliament currently debating about ways to strengthen the protection of academic freedom due to concerns that “[c]urrently, major breaches of and threats to academic freedom can be observed across Europe and the world” [17].

Yet, the relevance of academic freedom for innovation has never been empirically tested, to the best of our knowledge. To address this gap, we seek to provide an initial quantitative-empirical assessment of the relationship between academic freedom and innovation. To do so, we compile a comprehensive country-year dataset including 157 countries over the 1900–2015 period. We measure innovation output both in terms of *quantity*, proxied by the aggregate number of patent applications in per country and year, and *quality*, proxied by the aggregate number of forward citations per country and year. Annual data on country-level academic freedom come from the V-Dem Institute at the University of Gothenburg. The academic freedom index (AFI) provides a conceptually thorough, quantitative assessment of academic freedom [16], thereby enabling a comprehensive assessment of how academic freedom relates to innovation outcomes across countries and time. Overall, our sample (main analysis) covers up to 12,392 country-year observations, providing a exhaustive picture of inventive activity since the beginning of the 20^th^ century.

However, estimating the impact of academic freedom on innovation at the country level is not trivial. One potentially confounding explanation relates to spurious correlation. For example, rather than academic freedom, it might be that perceived freedom, correlated with both academic freedom and innovation output, is driving the association. Another potentially confounding explanation is reverse causality. That is, a country might adapt its level of academic freedom in response to an insufficient amount of past or contemporaneous innovation output. To overcome these threats of endogeneity, we implement a battery of instrumental variable tests. These approaches instrument current academic freedom with the level or stock of previous academic freedom or liberal democracy to address reverse causality and spurious correlation concerns, respectively. Instrumenting academic freedom with liberal democracy isolates the variation in academic freedom from the variation in general freedom and therefore uses as an estimator only the part of academic freedom that stems from academic freedom, addressing concerns about the perceived level of general freedom as a spurious confounder. Moreover, instrumenting current academic freedom with past academic freedom addresses reverse causality concerns if the lag is chosen for a long enough period.

Regardless of the empirical strategy chosen to identify causality, our results consistently suggest that academic freedom has strong positive effects on both the quantity and the quality of innovation output. First, an improvement in academic freedom by one standard deviation increases the number of patent applications two years later by 41%. The estimated effects for the instrumented academic freedom range from 37% to 61%, indicating that the estimated effect is robust. Second, an improvement in academic freedom by one standard deviation increases the number of forward citations five years later by 29%, and instrumented academic freedom yields effects of even larger magnitude. In post-hoc analyses, we show that these effects are largely robust to (1) the inclusion of additional country-level controls, (2) different of the dependent variables, (3) assessing only countries with high and low numbers of patent applications or forward citations, (4) assessing only the post-1980 period only to account for country-year panel imbalance, (5) using an alternative, de jure measure of academic freedom, (6) when examining various subdimensions of the academic freedom index, and (7) when account for time series properties.

We close by commenting that the positive relationship found between academic freedom and invention also means that the decline in academic freedom registered in the latest years could hinder the innovation rate and prosperity of countries in the years to come.

## 2. Background

### 2.1 Academic freedom

An abstract construct, academic freedom can be defined as “the right to choose one’s own problem for investigation, to conduct research free from any outside control, and to teach one’s subject in the light of one’s own opinions” [8: 583].

The institutionalization of the modern right to academic freedom took until the early 20^th^ century [18]. In the US, the focus of post-Civil War higher education shifted from preparing students for clergy and elite professions to training them for practical jobs, which resulted in researchers and teachers demanding independence from governing bodies. This led to the founding of the American Association of University Professors (AAUP) in 1915, which, together with the American Association of Colleges and Universities, published the Statement of Principles on Academic Freedom and Tenure in 1940, representing an important milestone for the establishment of academic freedom in the US. Today, the AAUP describes academic freedom as “the freedom of a teacher or researcher in higher education to investigate and discuss the issues in his or her academic field and to teach or publish findings without interference from political figures, boards of trustees, donors, or other entities. Academic freedom also protects the right of a faculty member to speak freely when participating in institutional governance, as well as to speak freely as a citizen” [19]. After World War II, the importance of academic freedom was reinstated, not only to ensure freedom of opinion from the influence of the political governments, but also as an ingredient necessary to unleash the full creative potential of science [18].

Today, the right to academic freedom is (legally) protected with considerable heterogeneity across jurisdictions. In the US, it is linked with the constitutional right of expression and speech in the First Amendment since the Supreme Court case of Sweezy v. New Hampshire (1959). However, the constitutional protection of academic freedom is limited to public universities, not to private institutions; pursuant to the State Action Doctrine and the First Amendment’s right of freedom of expression and speech, it does not cover all realms of academic freedom under the AAUP’s definition, such as the right to determine class curricula [19]. In the EU, the right of academic freedom is paragraphed in Article 13 of the Charter of Fundamental Rights, which states that the “arts and scientific research shall be free of constraint. Academic freedom shall be respected.” However, there is no common definition of academic freedom and member states have discretion over the enforcement of the right [20]. In China, academic freedom is deeply influenced by the country’s political structure, cultural heritage, and international aspirations. Lacking a legal foundation, academic freedom in China exists in a tension between the authoritarian nature of the CCP and the evolving internationalization of academia [21]. In India, academic freedom is indirectly protected by India’s constitution, which institutionalizes freedom of speech and expression. This freedom is subject to some limitations, which allows the state to impose reasonable restrictions on freedom of speech and expression in the interests of sovereignty, integrity, security of the state, public order, decency, or morality, or in relation to contempt of court, defamation, or incitement to an offense [22]. In Africa, some countries have specific references to academic freedom in their constitutions, offering a degree of legal protection, while others only provide indirect support through broader human rights or freedom of expression clauses [23].

**Fig 1:**
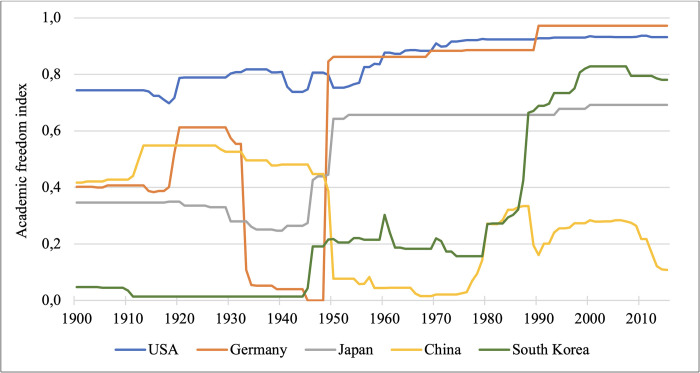
Academic freedom index over time (1900–2015) in selected countries. *Notes*: More information on the academic freedom index is provided in Table 1. Information for Germany is unavailable from 1945–1948.

To further illustrate the heterogeneity in academic freedom worldwide, Fig 1 plots the development of the academic freedom index from 1900 until 2015 for a selection of countries that currently produce a large number of patent applications per year (i.e., the US, Germany, China, Japan, South Korea)., Fig 1 illustrates considerable heterogeneity in the degree of academic freedom both between and within countries. Specifically, for Germany, the graph indicates a sharp decrease in academic freedom prior to World War II, followed by a substantial increase and subsequent stabilization at a high level (AFI information is unavailable from 1945–1948). The development of academic freedom in Japan mirrors that of Germany, but with post-World War II values stabilizing at a lower level. Fig 1 also shows that South Korea experienced a continuous growth in academic freedom and that the US maintained a consistently high level of academic freedom over the years. This contrasts with China, which experienced a marked decline in academic freedom, with current values far below Germany, the US, South Korea, and Japan.

### 2.2 Threats to academic freedom and recent decline

Mounting evidence suggests that academic freedom is currently under serious threat both in democratic and non-democratic countries. In 2023, the “Free to Think” annual report by Scholars at Risk’s Academic Freedom Monitoring Project lists 409 attacks on higher education (i.e., on scholars, students, institutions) that are “are indicative of deteriorating conditions for academic freedom”. These attacks include 161 cases of killings, violence, and disappearances, 86 cases of wrongful imprisonment and prosecution, and 46 cases of loss of position [24]. The attacks can come from a range of entities, such as “state and non-state actors, including armed militant and extremist groups, police and military forces, government authorities, off-campus groups, and even members of higher education communities” [22]. This is in line with longitudinal evidence suggesting a decline in global academic freedom over the last decade after many years of steady improvement [16]. The decline in academic freedom has provoked a resurge of the debate regarding the importance of academic freedom, for example, leading the European Parliament to bolster its legal protection in all EU Member States [17, 25].

Threats to academic freedom come from three main sources. First, interest groups moved by moral, religious, or ideological agendas voice concerns and attack professors on social media for their opinions, research, or teaching in controversial areas [26]. Examples include stem cell research, the use of animal models, and, more recently, views on the COVID-19 pandemic [27]. Second, some countries have regulations that allow political power to exert direct control over universities. For example, in China, department chairs and deans are centrally appointed, and a regulation that mandates a Party-appointed leader in all departments has been enforced since 2013 [28]. Finally, in some countries, the pursuit of for-profit opportunities in higher education institutions is progressively shifting the governance structures of academia, away from the collegiate model and toward a managerial model, which is more akin to a corporate research culture [27]. Universities become more responsive not only to policymakers but also to other stakeholders, who see the universities as service-providing organizations [29, 30]. This may induce academics to conform to institutional priorities and to eschew research themes that may be disliked by powerful donors and constituents, thus constraining research exploration and impact on academics’ integrity [31] and identity [32] with tension and moral conflict.

**Fig 2:**
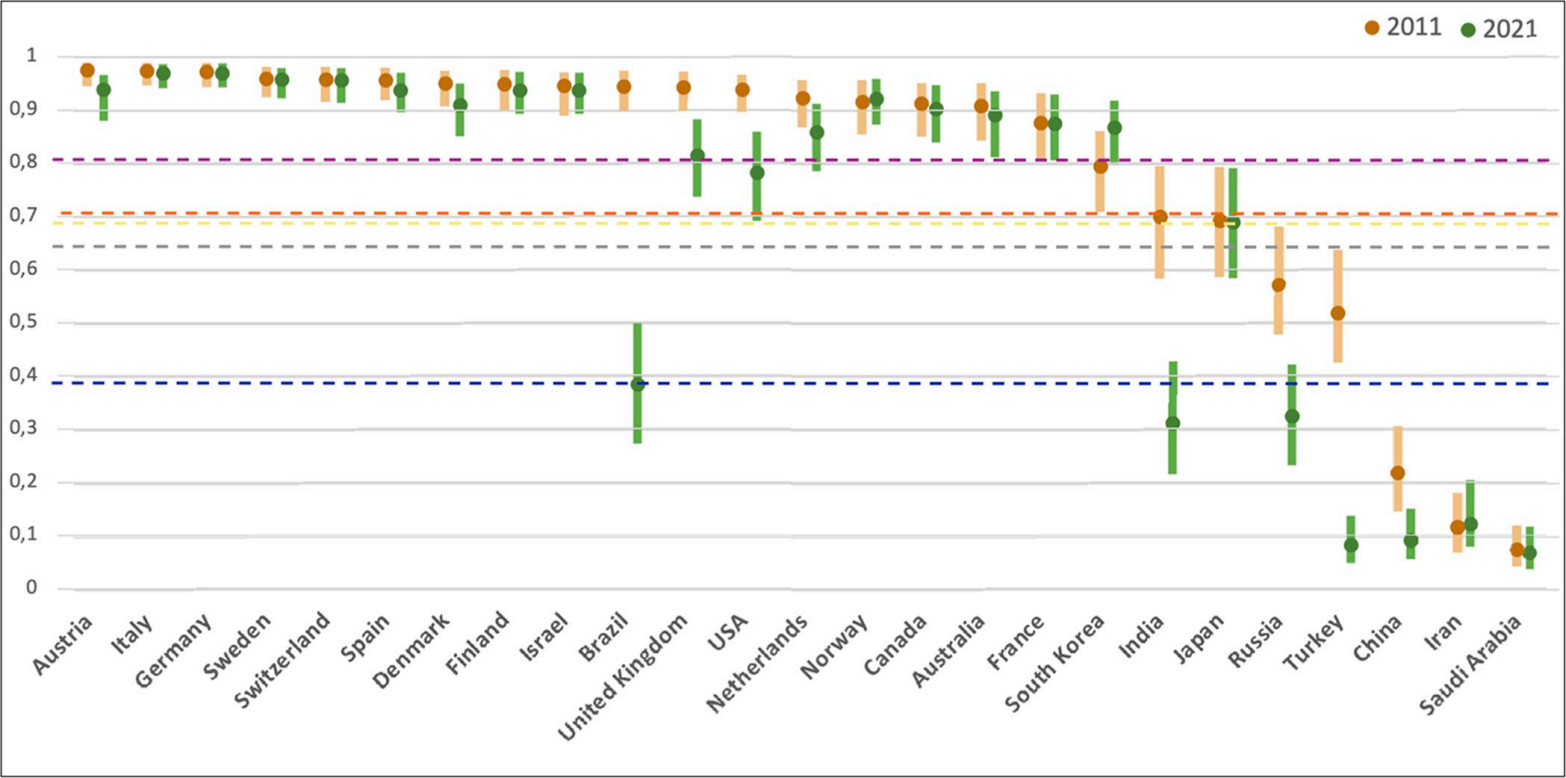
Academic freedom index in 2011 and 2021 in 25 leading countries by science base. *Notes*: Countries ordered in descending order of academic freedom index (AFI) in 2011. Interval bars represent min-max. Blue dashed line: average country level of AFI in 1941 (0.38). Gray dashed line: average country level of AFI in 1961 (0.65). Yellow dashed line: average country level of AFI in 1981 (0.69). Purple dashed line: average country level of AFI in 2001 (0.80). Red dashed line: average country level of AFI in 2021 (0.70). More information on the academic freedom index is provided in Table 1.

Against the background of these threats to academic freedom, research documents a concerning decline in academic freedom at the global level in the last decade, for the first time since World War II [16]. To substantiate these insights, we assess the subset of the 25 countries with the strongest science base, identified as the top-20 countries of the Scimago Country Ranking (1996–2021, all subject areas) either by H-Index, or by citations received (country ranking accessed on October 3^rd^, 2022 at: https://www.scimagojr.com/countryrank.php). Fig 2 reports the level of academic freedom registered in the 25 countries in 2011 and 2021. The graph shows a large heterogeneity across countries in 2021. Countries in Europe and North America, Australia, and South Korea have average AFI above 0.75. China, Iran, and Saudi Arabia have average AFI below 0.25. The comparisons of the mean country values in 2021 and 2011 indicate that only one country, South Korea, registered an improvement (+0.07) in the last decade. Fourteen countries remained overall stable (variations smaller than 0.02). Ten countries registered a reduction in academic freedom greater than -0.02. The most dramatic decreases were registered in Brazil (-0.56), Turkey (-0.43), India (-0.39) and Russia (-0.25). The USA (-0.15), UK (-0.13), and China (-0.12) also experienced a decrease. The average level of AFI across the 25 countries improved from 1941 to 2001, plateaued-off around 2001 to 2007, and declined afterward. The level of AFI in 2021 (0.70) is approximately equivalent to the one registered by the same countries in 1985.

### 2.3 Academic freedom and innovation

Freedom is seen as a source of growth and innovation since the beginning of modern economic thought [1, 2]. Also, institutional economics studies institutional design choices that enable free cooperation and competition to promote the production and exploitation of knowledge [3, 4]. Knowledge and innovation initiate spillover effects, which is a reason why freedom and innovation could be mutually reinforcing [33–35]. For example, the network theory of social capital argues that social freedom attracts talented people and promotes an atmosphere of tolerance, which fosters information exchange, knowledge spillovers, diversity and creativity, ultimately inducing innovative productivity [6].

To understand why academic freedom may relate to innovation, we must first explain why academia is important for innovation. Governments subsidize academics to invent because if inventing were left to the private sector alone, society would underinvest in innovative projects [9, 11]. One reason is that, given the existence of geographically bounded knowledge spillovers and the absence of perfect intellectual property rights, private agents would not be able to fully appropriate their inventions’ economic value.

The type of R&D that is best done by academia vis-à-vis industry illustrates why academic freedom plausibly spurs innovation. The optimal division of inventive effort between academia and industry is early-stage and late-stage R&D, respectively. One reason is that early-stage R&D is highly uncertain and therefore benefits from the inventors’ creative freedom to find a solution, while late-stage R&D is less uncertain and requires directed focus on monetizing an invention which is best achieved with hierarchy and delegated authority in the corporate setting [5]. Another reason is that the open exchange of ideas in academia facilitates knowledge spillovers but creates the risk of ideas being stolen. Instead, the control over information exchanges that occurs in corporate settings impedes knowledge spillovers and protects ideas from being stolen [36].

Both arguments build on the notion of academic freedom, which is a prerequisite for both creativity and open information exchange [37, 38]. Moreover, academia has various idiosyncratic norms, rules, and incentives that rely on academic freedom and that distinguish academics from corporate inventive efforts [39, 40]. Academic institutional features, such as the tenure system, peer review, and rewards for impactful work, promote the production and diffusion of knowledge [10, 41]. These institutional features work effectively only with a sufficiently high degree of academic freedom.

## 3. Data and variables

### 3.1 Data

We compile a longitudinal country-level dataset for 157 countries over the 1900–2015 period, merging data from three different sources. Our patent data comes from PATSTAT (2019 autumn edition), a comprehensive database on worldwide patent activity by the European Patent Office that is commonly used in innovation research [42, 43].

Data on academic freedom come of the V-Dem (Varierties of Democracy) dataset, which contains a wide array of indicators on democracy and related constructs, including academic freedom. The data is aggregated at the country-level and covers most countries in the world. Another feature is the dataset’s broad historical coverage going back as far as 1789 for some indicators. The V-Dem dataset is published by the V-Dem Institute based at the University of Gothenburg. The dataset is publicly available and updated regularly. We use data from the 2022 release (version 12). The academic freedom index is a well-developed indicator that is part of the V-Dem dataset since 2020. The academic freedom index was created in in collaboration between the V-Dem Institute and the University of Erlangen-Nuremberg (Germany). The index provides data for a large number of countries starting in 1900 and is described as the “first conceptually thorough assessment of academic freedom worldwide” [16: 3969].

Finally, we source some of our country-level controls from the 2022 World Population Prospects report by the United Nations.

### 3.2 Variables

#### 3.2.1 Dependent variables: Innovation quantity and quality

We study the effect of changes in academic freedom on innovation output, as represented by patents. Patents reflect the aggregate stock of technological knowledge in the economic that is tangible. Patenting activity is a standard proxy for innovation in innovation research [44]. We measure *innovation quantity* by the number of patent applications. Specifically, we consider only applications referring to invention patents (i.e., we exclude utility models and design patents) and count the number of applications filed in the focal country by application filing year. Regarding *innovation quality*, we consider the citations (i.e., forward citations) that all patent documents (applications and granted patents) received from all patent documents (applications and granted patents) of every origin within 3 years from the date of the deposit, using the procedure and query suggested by [45].

The final dataset comprises information on patent applications and forward citations related to the 157 countries for which the AFI is available and that were included in PATSTAT in the time window 1900–2015. Overall, our dataset covers up to 12,392 country-year observations, enabling a comprehensive account of inventive activity across countries and over time.

#### 3.2.2 Independent variable: Academic freedom index

Our main independent variable is the academic freedom index (AFI), which we source from the V-Dem dataset. Due to its extensive coverage and comprehensive measurement, the academic freedom index is a premier resource to assess differences in academic freedom across time and space [16].

The AFI is constructed via surveys of country experts that evaluate several subdimensions related to academic freedom. More specifically, more than 2,000 country experts (typically academics) were asked to provide their evaluation of five indicators [46]: (1) freedom to research and teach, (2) freedom of academic exchange and dissemination, (3) institutional autonomy, (4) campus integrity, and (5) freedom of academic and cultural expression. For example, the subdimension (1) freedom of academic and cultural expression was captured via the question: “To what extent are scholars free to develop and pursue their own research and teaching agendas without interference?”. The response options were: “0: Completely restricted. When determining their research agenda or teaching curricula, scholars are, across all disciplines, consistently subject to interference or incentivized to self-censor.”, “1: Severely restricted. When determining their research agenda or teaching curricula, scholars are, in some disciplines, consistently subject to interference or incentivized to self-censor.” “2: Moderately restricted. When determining their research agenda or teaching curricula, scholars are occasionally subject to interference or incentivized to self-censor.” “3: Mostly free. When determining their research agenda or teaching curricula, scholars are rarely subject to interference or incentivized to self-censor.” “4: Fully free. When determining their research agenda or teaching curricula, scholars are not subject to interference or incentivized to self-censor.” The country experts were asked to provide scores from 1900 onwards. The independent expert opinions are calibrated and aggregated by point estimates drawn from a Bayesian factor analysis aimed at minimizing potential biases due to multiple-raters and issues of consistency across countries and years. The individual subdimensions are the combined into the academic freedom index, which quantifies the degree to which academic freedom is respected in a given country and year, on an interval scale from 0 (low degree of academic freedom) to 1 (high degree of academic freedom).

The V-Dem institute publishes a detailed codebook [46] and methodology guide [47] that provide detailed insights into the construction of the AFI and include all questions used. Additionally, the AFI’s validity is assessed and documented by [16].

#### 3.2.3 Other variables

Our baseline estimations include country and year dummies to capture time-variant and between-country confounding factors. We also employ several instrumental variables for academic freedom and show that our results are robust to various modifications to our baseline estimation, such as the inclusion of additional controls, such as GDP per capita, population size, and migration rate.

## 4. Results

### 4.1 Summary statistics

Summary statistics and descriptions of our variables are reported in Table 1. The number of patent applications (natural logarithm, leaded by two years) is on average 1.908 with a standard deviation of 3.206. Without the log-transformation, the average country in our sample files 3,449 (standard deviation = 28,948) patent applications per year. On average, these patents receive 2,072 forward citations over the next three years with a standard deviation of 39,625. There is substantial positive skewness in these variables, given that the minimum is at 0 and the maximum is at around 1.2 million for the number of patent applications. The same is true for the citation data, which ranges from 0 to 1.3 million. This highlights the need for a log-transformation and for the inclusion of country dummies in our empirical models.

**Table 1:** Descriptive statistics and definition of variables.

Variable	Obs. (countries)	Mean (SD)	SD between, SD within	Min., Max.	Description	Source
*Dependent variables*:						
Patent applications_t+2_ (ln)	18,212 (157)	1.908 (3.206)	2.634, 1.840	0.000, 14.138	Number of patent applications referring to invention patents per country/year.	PATSTAT
Forward citationst+5 (ln)	17,741 (157)	0.440 (1.633)	1.145, 1.168	0.000, 14.129	Number of forward citations that all patent documents (applications and granted patents) received from all patent documents (applications and granted patents) of every origin within 3 years from the date of the deposit (per country/year).	PATSTAT
*Independent variable*						
Academic freedom (z)	12,706 (157)	0.000 (1.000)	0.811, 0.624	-1.620, 1.476	Degree of academic freedom per country/year.	V-Dem
*Instruments*						
Democracy_t-5_ (z)	15,002 (157)	0.000 (1.000)	0.800, 0.607	-0.986, 2.546	Degree to which the ideal of liberal democracy achieved per country/year.	V-Dem
Democracy stock_t-10_ (z)	14,081 (157)	0.000 (1.000)	0.835, 0.575	-1.006, 2.534	Stock variable that captures the degree to which the ideal of liberal democracy achieved per country/year over the previous 10 years with a deprecation rate of 1%.	V-Dem
AF stock_t-10_ (z)	11,071 (156)	0.000 (1.000)	0.848, 0.590	-1.634, 1.522	Stock variable that captures the degree of academic freedom per country/year over the previous 10 years with a deprecation rate of 1%.	V-Dem
*Variables used in robustness checks*						
GDP per capita	13,859 (156)	8.655 (12.524)	11.350, 7.762	0.286, 156.628	Gross domestic product on a per capita basis per country/year.	V-Dem
Population (ln)	10,200 (150)	8.759 (1.721)	1.668, 0.444	3.174, 14.156	Total population per country/year.	UN
Migration rate	10,200 (150)	0.526 (14.835)	7.227, 12.969	-526.323, 415.239	Net migration rate (per 1,000 population) per country/year	UN
AF: de jure	10,571 (156)	0.000 (1.000)	0.795, 0.638	0.752, 1.329	Dummy that captures whether constitutional provisions for the protection of academic freedom exist.	V-Dem
AF: freedom to research and teach (z)	12,701 (157)	0.000 (1.000)	0.804, 0.640	-2.563, 2.060	Degree to which scholars are free to develop and pursue their own research and teaching agendas without interference.	V-Dem
AF: freedom of academic exchange and dissemination (z)	12,701 (157)	0.000 (1.000)	0.802, 0.632	-2.508, 1.909	Degree to which are scholars free to exchange and communicate research ideas and findings	V-Dem
AF: institutional autonomy (z)	12,762 (157)	0.000 (1.000)	0.813, 0.644	-2.604, 2.064	Degree to which universities exercise institutional autonomy in practice.	V-Dem
AF: campus integrity (z)	12,708 (157)	0.000 (1.000)	0.820, 0.605	-2.352, 1.958	Degree to which campuses are free from politically motivated surveillance or security infringements.	V-Dem
AF: freedom of academic and cultural expression (z)	16,105 (157)	0.000 (1.000)	0.731, 0.695	-2.201, 2.252	Degree to which scholars and university students publicly criticize government policies.	V-Dem

*Notes*: SD = standard deviation, (ln) = variable is log-transformed. (z) = variable is z-standardized. Sources: PATSTAT = PATSTAT database by the European Patent Office (2019 autumn edition, for further information, see https://www.epo.org/searching-for-patents/business/patstat.html, last accessed: 6 February 2024), V-Dem = Varieties of Democracy dataset (version: 12, available at: https://v-dem.net/data/dataset-archive/, last accessed: 6 February 2024), UN = United Nations World Population Prospects 2022 (version: July 2022, available at https://population.un.org/wpp/, last accessed: 6 February 2024). The number of observations used in the multivariate analyses varies based on the availability of data per country/year.

The academic freedom index is z-standardized (mean = 0, standard deviation = 1). The mean of the unstandardized variable is 0.516 (scaled from 0 to 1), indicating a medium level of academic freedom across countries and years.

The average country-year observation in our sample has a GDP per capita of $8,655, a population of 30.6 million inhabitants (mean of the log-transformed variable = 8.759), and a migration rate of 0.526.

### 4.2 Identification strategy to mitigate endogeneity concerns

Our baseline models regress the two country-year innovation output proxies (i.e., patent applications and forward citations) on the AFI, while also including country and year dummies. This specification may incur two specific concerns about its ability to identify whether any estimated association between academic freedom and innovation output is causal. First, a concern with the baseline specification is that there might be other, omitted factors, such as the level of general freedom in a country, that are correlated with both academic freedom and innovation output (spurious correlation). Second, a country may adjust its level of academic freedom in response to the state of the country’s current innovation output (reverse causality). Specifically, instead of academic freedom driving innovation output, the concern is that innovation output (or a relative lack thereof) may drive academic freedom.

To address these concerns, we implement four distinct instrumental variable approaches from the political analysis literature [48–50]. First, we instrument academic freedom with the country’s five-year-lagged liberal democracy level (approach 1) and with the country’s liberal democracy stock calculated over the previous ten years with a 1% deprecation rate (approach 2). Approaches 1 and 2 address concerns about spurious correlations (e.g., that the general level of freedom could confound the measured effect of academic freedom on the innovative performance in a country) and instrument academic freedom with an index of country-year liberal democracy, following [48]. The intuition behind the instrument is that liberal democracies impact academic freedom because the more liberal rights in a society, the higher the likelihood that the right to conduct research and teach free of any restrictions. In econometric terms, this satisfies the relevance restriction. Regarding the exclusion restriction, research in innovation management suggests the democracy does not affect innovation directly, which could indicate that democracy affects innovation via academic freedom. However, the democracy-innovation debate is ongoing, and there is also contradictory evidence suggesting a direct link between democracy and innovation [51].

Second, we instrument academic freedom with the country’s five-year-lagged AFI, assuming that reverse causality concerns do not exist for more than five years (approach 3), and with the country’s academic freedom stock calculated over the previous ten years with a depreciation rate of 1% (approach 4). Approaches 3 and 4 use values of the AFI in previous years as an instrument to address concerns of reverse causality (e.g., that the inventiveness of a country induces more academic freedom). These approaches of using lagged instruments rely on the argument of rational expectations in economics [52].

### 4.3 Main results

#### 4.3.1 Academic freedom and innovation quantity

Table 2 shows the regression results for innovation quantity. The dependent variable is the natural logarithm of the two-year-leaded aggregate number of patent applications per year and country. The independent variable in Model 1 is the z-standardized AFI per country and year. The independent variables in Models 2, 3, 4, and 5 are the instrumented versions of the AFI described before. The number of country-year observations ranges from 10,753 to 12,392 across model specifications due to limited availabilities of the instruments for some country-years. The (adjusted) R-squared of our models exceeds 80%, indicating that our models capture a substantial of the variation in innovation output in terms of aggregate country-level patent applications.

**Table 2:** Main analysis of the effect of academic freedom (AF) on innovation quantity (patent applications).

Model	(1)		(2)		(3)		(4)		(5)	
Dependent variable	*Patent applications*_*t+2*_ *(ln)*		*Patent applications*_*t+2*_ *(ln)*		*Patent applications*_*t+2*_ *(ln)*		*Patent applications*_*t+2*_ *(ln)*		*Patent applications*_*t+2*_ *(ln)*	
Statistic	*Coeff*.	*(SE)*	*Coeff*.	*(SE)*.	*Coeff*.	*(SE)*	*Coeff*.	*(SE)*	*Coeff*.	*(SE)*
*Indepdent variables*										
AF (z)	0.345	(0.032)***								
AF (IV: Democracy_t-5_, z)			0.477	(0.060)***						
AF (IV: Democracy stock_t-10_, z)					0.442	(0.057)***				
AF (IV: AF_t-5_, z)							0.330	(0.043)***		
AF (IV: AF stock_t-10_, z)									0.315	(0.042)***
*Controls*										
Year/country dummies	Yes		Yes		Yes		Yes		Yes	
Countries (observations)	157	(12,392)	157	(11,809)	157	(11,257)	157	(11,569)	156	(10,753)
R^2^ (R^2^ adjusted)	0.811	(0.807)	0.815	(0.811)	0.818	(0.814)	0.817	(0.812)	0.820	(0.816)
MOP weak instrument test (F)	-		2,395.13	(τ < 0.05)	4,114.46	(τ < 0.05)	5,946.18	(τ < 0.05)	11,895.90	(τ < 0.05)

*Notes*: The estimates in Model (1) are obtained from a pooled OLS regression. The estimates in Models (2)–(5) come from 2SLS regressions. All variables are described in Table 1. Robust standard errors in parentheses. The MOP weak instrument is based on Montiel Olea and Pflueger (2013), as implemented in STATA’s weakivtest command. High F-values indicate a strong instrument.

* p < 0.10

** p < 0.05

*** p < 0.01.

Instrument statistics show that lagged and stock liberal democracy and lagged and stock academic freedom are strong instruments. The weak-instrument test by [53] shows a large F statistic and rejects the null hypothesis that our instruments of academic freedom are weak.

The regression results across all models show a positive and statistically significant (p < .01) effect of academic freedom on patent applications, which suggests that an increase (decrease) in academic freedom leads to an increase (decrease) in the quantity of innovation output. The baseline (uninstrumented, pooled OLS) estimation in Model (1) yields a coefficient of the z-standardized AFI of 0.345 (robust standard error = 0.032, p < .01), suggesting that a one standard deviation increase in academic freedom increases the average number of patent applications by 41.2% (= exp(0.345)-1). The 2SLS results in Models (2) to (5) are consistent with the result in Model (1), indicating that academic freedom has a positive effect on the number of patent applications. For all four instrumented versions of academic freedom, we estimate coefficients in the range of 0.315 and 0.477 (p < .01) Therefore, depending on the instrument, improving academic freedom by one standard deviation translates into 37.0% to 61.1% more patent applications two years later.

#### 4.3.2 Academic freedom and innovation quality

Table 3 shows regression results for innovation quality. The dependent variable is the natural logarithm of the five year-leaded aggregate number of forward citations per year and country. The independent variables are the same as those in Table 2. The only difference (in addition to changing the dependent variable) is the inclusion of the natural logarithm of the number of patent applications as a control variable. Conditioning on the number of patent applications helps ensure that any identified effect on the quality of innovation output is indeed due to academic freedom and not through a mediating mechanism (i.e., academic freedom increases patent applications and, therefore, also forward citations). Our model captures large amounts of variation in the dependent variable, with R-squares always larger than 60%. Moreover, the weak-instrument test by [53] shows indicates that our instrument is not weak.

**Table 3:** Main analysis of the effect of academic freedom (AF) on innovation quality (forward citations received in the first 3 years).

Model	(1)		(2)		(3)		(4)		(5)	
Dependent variable	*Forward citations*_*t+5*_ *(ln)*		*Forward citations*_*t+5*_ *(ln)*		*Forward citations*_*t+5*_ *(ln)*		*Forward citations*_*t+5*_ *(ln)*		*Forward citations*_*t+5*_ *(ln)*	
Statistic	*Coeff*.	*(SE)*	*Coeff*.	*(SE)*	*Coeff*.	*(SE)*	*Coeff*.	*(SE)*	*Coeff*.	*(SE)*
*Indepdenent variables*										
AF (z)	0.258	(0.022)***								
AF (IV: Democracy_t-5_, z)			1.149	(0.057)***						
AF (IV: Democracy stock_t-10_, z)					1.126	(0.053)***				
AF (IV: AF_t-5_, z)							0.431	(0.031)***		
AF (IV: AF stock_t-10_, z)									0.436	(0.031)***
*Controls*										
Patent applications (ln)	Yes		Yes		Yes		Yes		Yes	
Year/country dummies	Yes		Yes		Yes		Yes		Yes	
Countries (observations)	157	(11,921)	157	(11,338)	157	(10,786)	156	(11,099)	156	(10,285)
R^2^ (R^2^ adjusted)	0.642	(0.633)	0.607	(0.598)	0.631	(0.621)	0.659	(0.650)	0.680	(0.671)
MOP weak instrument test (F)	-		2,114.24	(τ < 0.05)	3,610.46	(τ < 0.05)	5,313.93	(τ < 0.05)	10,607.19	(τ < 0.05)

*Notes*: The estimates in Model (1) are obtained from a pooled OLS regression. The estimates in Models (2)–(5) come from 2SLS regressions. All variables are described in Table 1. Robust standard errors in parentheses. The MOP weak instrument is based on [53], as implemented in STATA’s weakivtest command. High F-values indicate a strong instrument.

* p < 0.10

** p < 0.05

*** p < 0.01.

The baseline regression estimates a coefficient of 0.258 (robust standard error = 0.022, p < .01) in Model (1). Therefore, improving academic freedom by one standard deviation translates into more forward citations in the amount of 29.4% (= exp(0.258)-1). The four instrumented versions suggest that academic freedom’s causal effect on forward citations is even higher. The estimated coefficients in the 2SLS specifications range from 0.431 to 1.149 (p < .01). Depending on the specification, this suggests that a once standard deviation increase in academic freedom can be associated with an increase in forward citations by 53.9% to 215.5%.

### 4.4 Robustness checks

We estimate a series of additional models to assess the robustness of our results, which we briefly motivate and describe below.

#### 4.4.1 Additional control variables

While the year and country dummies capture a substantial amount of variance in our dependent variables, we reestimate our main models with additional time-varying control variables (i.e., GDP growth, population (ln), and migration rate).

The results are displayed in Table 4 (innovation quantity) and Table 5 (innovation quality). While the results show associations between the control variables and our innovation measures, the main effect of AFI on innovation output remains positive and statistically significant (p < .01) across all specifications. This underlines the robustness of our main results.

**Table 4:** The effect of academic freedom (AF) on innovation output (patent applications) with additional control variables.

Model	(1)		(2)		(3)		(4)		(5)	
Dependent variable	*Patent applications*_*t+2*_ *(ln)*		*Patent applications*_*t+2*_ *(ln)*		*Patent applications*_*t+2*_ *(ln)*		*Patent applications*_*t+2*_ *(ln)*		*Patent applications*_*t+2*_ *(ln)*	
Statistic	*Coeff*.	*(SE)*	*Coeff*.	*(SE)*	*Coeff*.	*(SE)*	*Coeff*.	*(SE)*	*Coeff*.	*(SE)*
*Indepedent variables*										
AF (z)	0.275	(0.037)***								
AF (IV: Democracy_t-5_, z)			0.221	(0.070)***						
AF (IV: Democracy stock_t-10_, z)					0.250	(0.065)***				
AF (IV: AF_t-5_, z)							0.316	(0.053)***		
AF (IV: AF stock_t-10_, z)									0.305	(0.052)***
*Control variables*										
GDP per capita	-0.005	(0.004)	-0.004	(0.004)	-0.004	(0.004)	-0.005	(0.005)	-0.007	(0.005)
Population (ln)	0.112	(0.113)	0.148	(0.124)	0.166	(0.125)	0.216	(0.119)*	0.301	(0.125)*
Migration rate	0.001	(0.001)*	0.001	(0.001)*	0.001	(0.001)*	0.001	(0.001)*	0.001	(0.001)*
Year/country dummies	Yes		Yes		Yes		Yes		Yes	
Countries (observations)	148	(7,257)	148	(7,092)	148	(6,962)	148	(6,980)	147	(6,679)
R^2^ (R^2^ adjusted)	0.870	(0.866)	0.870	(0.866)	0.870	(0.866)	0.871	(0.867)	0.871	(0.867)

*Notes*: The estimates in Model (1) are obtained from a pooled OLS regression. The estimates in Models (2)–(5) come from 2SLS regressions. All variables are described in Table 1. Robust standard errors in parentheses.

* p < 0.10

** p < 0.05

*** p < 0.01.

**Table 5:** The effect of academic freedom (AF) on innovation output (forward citations) with additional control variables.

Model	(1)		(2)		(3)		(4)		(5)	
Dependent variable	*Forward citations*_*t+5*_ *(ln)*		*Forward citations*_*t+5*_ *(ln)*		*Forward citations*_*t+5*_ *(ln)*		*Forward citations*_*t+5*_ *(ln)*		*Forward citations*_*t+5*_ *(ln)*	
Statistic	*Coeff*.	*(SE)*	*Coeff*.	*(SE)*	*Coeff*.	*(SE)*	*Coeff*.	*(SE)*	*Coeff*.	*(SE)*
*Indepedent variables*										
AF (z)	0.193	(0.022)***								
AF (IV: Democracy_t-5_, z)			0.494	(0.055)***						
AF (IV: Democracy stock_t-10_, z)					0.516	(0.053)***				
AF (IV: AF_t-5_, z)							0.356	(0.033)***		
AF (IV: AF stock_t-10_, z)									0.390	(0.033)***
*Control variables*										
GDP per capita	0.037	(0.003)***	0.040	(0.004)***	0.044	(0.004)***	0.046	(0.004)***	0.053	(0.004)***
Population (ln)	-0.928	(0.066)***	-0.803	(0.076)***	-0.792	(0.078)***	-0.867	(0.074)***	-0.855	(0.084)***
Migration rate	-0.002	(0.001)***	-0.002	(0.001)***	-0.002	(0.001)***	-0.002	(0.001)***	-0.003	(0.001)***
Patent applications (ln)	Yes		Yes		Yes		Yes		Yes	
Year/country dummies	Yes		Yes		Yes		Yes		Yes	
Countries (observations)	149	(7,761)	149	(7,586)	149	(7,428)	148	(7,431)	148	
R^2^ (R^2^ adjusted)	0.871	(0.867)	0.867	(0.864)	0.868	(0.864)	0.872	(0.868)		

*Notes*: The estimates in Model (1) are obtained from a pooled OLS regression. The estimates in Models (2)–(5) come from 2SLS regressions. All variables are described in Table 1. Robust standard errors in parentheses.

* p < 0.10

** p < 0.05

*** p < 0.01.

#### 4.4.2 Different lags of the dependent variables

In our main analysis, our dependent variable is forwarded by two years (innovation quantity) and five years (innovation quality). This is equal to lagging all variables except for the dependent variable by two respectively five years. We introduce this lag structure because we do not expect changes in academic freedom to have an immediate effect on innovation output and may take several years to manifest. Because the choice of lag structure is somewhat arbitrary, especially when considering many countries across a considerable period of time, we assess whether different time windows affect our estimates in a robustness check.

The results in Tables 6 and 7 show that the effect of academic freedom on innovation quantity and quality remains positive and statistically significant (p < .01) when considering innovation output forwarded by 0 to 4 years.

**Table 6:** The effect of academic freedom (AF) on innovation output (patent applications) when considering different lags of the dependent variable.

Model	(1)		(2)		(3)		(4)		(5)	
Dependent variable	*Patent applications*_*t*_ *(ln)*		*Patent applications*_*t+1*_ *(ln)*		*Patent applications*_*t+2*_ *(ln)*		*Patent applications*_*t+3*_ *(ln)*		*Patent applications*_*t+4*_ *(ln)*	
Statistic	*Coeff*.	*(SE)*	*Coeff*.	*(SE)*.	*Coeff*.	*(SE)*	*Coeff*.	*(SE)*	*Coeff*.	*(SE)*
*Independent variable*										
AF (z)	0.335	(0.032)***	0.343	(0.032)***	0.345	(0.032)***	0.336	(0.032)***	0.327	(0.033)***
*Controls*										
Year/country dummies	Yes		Yes		Yes		Yes		Yes	
Countries (observations)	157	(12,706)	157	(12,549)	157	(12,392)	157	(12,235)	157	(12,078)
R^2^ (R^2^ adjusted)	0.807	(0.803)	0.809	(0.805)	0.811	(0.807)	0.812	(0.807)	0.812	(0.808)

*Notes*: The estimates are obtained from pooled OLS regressions. All variables are described in Table 1. Robust standard errors in parentheses.

* p < 0.10

** p < 0.05

*** p < 0.01.

**Table 7:** The effect of academic freedom (AF) on innovation output (forward citations) when considering different lags of the dependent variable.

Model	(1)		(2)		(3)		(4)		(5)	
Dependent variable	*Forward citations*_*t*_ *(ln)*		*Forward citations*_*t+1*_ *(ln)*		*Forward citations*_*t+2*_ *(ln)*		*Forward citations*_*t+3*_ *(ln)*		*Forward citations*_*t+4*_ *(ln)*	
Statistic	*Coeff*.	*(SE)*	*Coeff*.	*(SE)*.	*Coeff*.	*(SE)*	*Coeff*.	*(SE)*	*Coeff*.	*(SE)*
*Independent variable*										
AF (z)	0.174	(0.022)***	0.192	(0.022)***	0.210	(0.022)***	0.228	(0.022)***	0.245	(0.022)***
*Controls*										
Patent applications (ln)	Yes		Yes		Yes		Yes		Yes	
Year/country dummies	Yes		Yes		Yes		Yes		Yes	
Countries (observations)	157	(12,706)	157	(12,549)	157	(12,392)	157	(12,235)	157	(12,078)
R^2^ (R^2^ adjusted)	0.616	(0.608)	0.621	(0.613)	0.626	(0.618)	0.631	(0.623)	0.636	(0.628)

*Notes*: The estimates are obtained from pooled OLS regressions. All variables are described in Table 1. Robust standard errors in parentheses.

* p < 0.10

** p < 0.05

*** p < 0.01.

#### 4.4.3 Sample split according to innovation output

For some countries in our dataset, PATSTAT did not record any patent applications or forward citations. To assess the existence of related biases (e.g., countries not reporting their patents, data issues in PATSTAT), we perform multiple sample splits to assess whether the relation between academic freedom and innovation outcomes holds across groups of countries that substantially differ in their innovation output. Specifically, we calculate the total number of patents application and forward citations per country, and then rank the countries accordingly. We then reestimate our main models based on the subsamples of the top 25 countries, the top 50 countries, the top 75 countries (only for innovation quantity), the remaining countries not part of the top 50 (innovation quality) or 75 (innovation quantity), and when only considering countries with non-zero numbers of patent applications or forward citations. Notice that these subsamples are not mutually exclusive (e.g., the top 25 countries are also part of the top 50 countries).

The results are displayed in Table 8 (innovation quantity) and Table 9 (innovation quality). For both quantity and quality, the estimates show that the effect of academic freedom on innovation quantity is pronounced and positive in countries with a high number of patent applications, but is less pronounced and loses statistical significance when only considering countries with lower innovation output.

**Table 8:** The effect of academic freedom (AF) on innovation output (patent applications) with the sample split according to the total number of patent applications per country.

**Model**	**(1)**		**(2)**		**(3)**		**(4)**		**(5)**	
**Sample**	*Top 25 countries*		*Top 50 countries*		*Top 75 countries*		*Countries not in top 75*		*Excluding countries with 0 patents*	
**Dependent variable**	*Patent applications*_*t+2*_ *(ln)*		*Patent applications*_*t+2*_ *(ln)*		*Patent applications*_*t+2*_ *(ln)*		*Patent applications*_*t+2*_ *(ln)*		*Patent applications*_*t+2*_ *(ln)*	
**Statistic**	*Coeff*.	*(SE)*	*Coeff*.	*(SE)*.	*Coeff*.	*Coeff*.	*Coeff*.	*(SE)*	*Coeff*.	*(SE)*
*Independent varibale*										
AF (z)	0.765	(0.089)***	0.441	(0.051)***	0.398	(0.040)***	-0.057	(0.019)***	0.336	(0.033)***
*Controls*										
Year/country dummies	Yes		Yes		Yes		Yes		Yes	
Countries (observations)	25	(2,799)	50	(5,344)	75	(7,172)	82	(5,220)	142	(11,668)
R^2^ (R^2^ adjusted)	0.683	(0.666)	0.759	(0.751)	0.781	(0.775)	0.491	(0.471)	0.807	(0.803)

*Notes*: The estimates are obtained from pooled OLS regressions. All variables are described in Table 1. Robust standard errors in parentheses.

* p < 0.10

** p < 0.05

*** p < 0.01.

**Table 9:** The effect of academic freedom (AF) on innovation output (forward citations) with the sample split according to the total number of forward citations per country.

Model	(1)		(2)		(3)		(4)	
Sample	*Top 25 countries*		*Top 50 countries*		*Countries not in top 50*		*Excluding countries with 0 citations*	
Dependent variable	*Forward citations*_*t+5*_ *(ln)*		*Forward citations*_*t+5*_ *(ln)*		*Forward citations*_*t+5*_ *(ln)*		*Forward citations*_*t+5*_ *(ln)*	
Statistic	*Coeff*.	*(SE)*	*Coeff*.	*(SE)*.	*Coeff*.	*(SE)*	*Coeff*.	*(SE)*
*Independent varibale*								
AF (z)	0.399	(0.063)***	0.362	(0.042)***	-0.001	(0.002)	0.395	(0.032)***
*Controls*								
Patent applications (ln)	Yes		Yes		Yes		Yes	
Year/country dummies	Yes		Yes		Yes		Yes	
Countries (observations)	25	(2,664)	51	(4,943)	106	(6,978)	73	(6,576)
R^2^ (R^2^ adjusted)	0.756	(0.743)	0.681	(0.670)	0.165	(0.138)	0.653	(0.643)

*Notes*: The estimates are obtained from pooled OLS regressions. All variables are described in Table 1. Robust standard errors in parentheses.

* p < 0.10

** p < 0.05

*** p < 0.01.

#### 4.4.4 Only considering years after 1980

While data on academic freedom is available for most countries from 1900 onwards, patent data available in PATSTAT is less complete. While countries like the US show meaningful patent activity in PATSTAT since the end of the 19^th^ century, many countries included in PATSTAT did not report any patents until much later. This is illustrated by the European Patent Office’s overview of the data included in the 2018 version of PATSTAT. There are multiple plausible reasons for the lack of data in PATSTAT, such as the absence of a patent system in some countries, the lack of a systematic data collection on patents, or the lack of a transfer of the data to the EPO for the inclusion in PASTAT. While we code these entries as 0 in our main analysis, we perform a robustness check in which we only consider years after 1980, after which coverage improved.

The results are displayed in Table 10 (innovation quantity) and Table 11 (innovation quality). While the number of observations is significantly lower than in our main analysis, the effect of academic freedom on innovation outcomes remains positive and statistically significant (p < 0.01) across all specifications, underlining the robustness of our results.

**Table 10:** The effect of academic freedom (AF) on innovation output (patent applications) when only considering years after 1980.

Model	(1)		(2)		(3)		(4)		(5)	
Dependent variable	*Patent applications*_*t+2*_ *(ln)*		*Patent applications*_*t+2*_ *(ln)*		*Patent applications*_*t+2*_ *(ln)*		*Patent applications*_*t+2*_ *(ln)*		*Patent applications*_*t+2*_ *(ln)*	
Statistic	*Coeff*.	*(SE)*	*Coeff*.	*(SE)*	*Coeff*.	*(SE)*	*Coeff*.	*(SE)*	*Coeff*.	*(SE)*
*Independent variables*										
AF (z)	0.311	(0.060)***								
AF (IV: Democracy_t-5_, z)			0.626	(0.105)***						
AF (IV: Democracy stock_t-10_, z)					0.682	(0.096)***				
AF (IV: AF_t-5_, z)							0.578	(0.083)***		
AF (IV: AF stock_t-10_, z)									0.567	(0.079)***
*Controls*										
Year/country dummies	Yes		Yes		Yes		Yes		Yes	
Countries (observations)	157	(5,190)	157	(5,042)	157	(4,924)	157	(5,046)	156	(4,892)
R^2^ (R^2^ adjusted)	0.919	(0.916)	0.920	(0.917)	0.920	(0.917)	0.920	(0.917)	0.921	(0.917)

*Notes*: The estimates are obtained from pooled OLS regressions. All variables are described in Table 1. Robust standard errors in parentheses.

* p < 0.10

** p < 0.05

*** p < 0.01.

**Table 11:** The effect of academic freedom (AF) on innovation output (forward citations) when only considering years after 1980.

Model	(1)		(2)		(3)		(4)		(5)	
Dependent variable	*Forward citations*_*t+5*_ *(ln)*		*Forward citations*_*t+5*_ *(ln)*		*Forward citations*_*t+5*_ *(ln)*		*Forward citations*_*t+5*_ *(ln)*		*Forward citations*_*t+5*_ *(ln)*	
Statistic	*Coeff*.	*(SE)*	*Coeff*.	*(SE)*	*Coeff*.	*(SE)*	*Coeff*.	*(SE)*	*Coeff*.	*(SE)*
*Independent variables*										
AF (z)	0.137	(0.033)***								
AF (IV: Democracy_t-5_, z)			0.637	(0.086)***						
AF (IV: Democracy stock_t-10_, z)					0.650	(0.083)***				
AF (IV: AF_t-5_, z)							0.335	(0.052)***		
AF (IV: AF stock_t-10_, z)									0.372	(0.051)***
*Controls*										
Patent applications (ln)	Yes		Yes		Yes		Yes		Yes	
Year/country dummies	Yes		Yes		Yes		Yes		Yes	
Countries (observations)	157	(4,719)	157	(4,571)	157	(4,453)	156	(4,576)	156	(4,424)
R^2^ (R^2^ adjusted)	0.913	(0.909)	0.907	(0.903)	0.907	(0.903)	0.913	(0.909)	0.912	(0.909)

*Notes*: The estimates are obtained from pooled OLS regressions. All variables are described in Table 1. Robust standard errors in parentheses.

* p < 0.10

** p < 0.05

*** p < 0.01.

#### 4.4.5 Using a de jure measure of academic freedom

A difference exists between *de jure* and *de facto* academic freedom. While de jure academic freedom refers to an institutionally allocated level of academic freedom, de facto academic freedom reflects the actual level of academic freedom in a society and can differ from the de jure level [54]. For example, many women were excluded from important academic institutions until recently (in some places they are still excluded), even though they are formally allowed to participate in academia. While academic freedom existed in a de jure sense, many people were de facto excluded from this freedom, with considerable economic and social costs. Generally, many groups, and sometimes entire populations, are informally denied the freedom to engage in research that they want and are formally allowed to pursue.

Hence, Table 12 (innovation quantity) and Table 13 (innovation quality) employ an alternative measure for academic freedom that captures whether academic freedom was constitutionally protected in a given country year. Like our de facto measure of academic freedom, this de jure measure comes from the V-Dem Institute’s academic freedom dataset, in which country experts were asked to respond to the question: “Do constitutional provisions for the protection of academic freedom exist?” (0 = no, 1 = yes). Overall, the positive association between academic freedom and innovation output holds (p < 0.01) when the de jure measure is considered.

**Table 12:** The effect of academic freedom (AF) on innovation output (patent applications) with a *de jure* metric of AF.

Model	(1)		(2)		(3)		(4)		(5)	
Dependent variable	*Patent applications*_*t+2*_ *(ln)*		*Patent applications*_*t+2*_ *(ln)*		*Patent applications*_*t+2*_ *(ln)*		*Patent applications*_*t+2*_ *(ln)*		*Patent applications*_*t+2*_ *(ln)*	
Statistic	*Coeff*.	*(SE)*	*Coeff*.	*(SE)*	*Coeff*.	*(SE)*	*Coeff*.	*(SE)*	*Coeff*.	*(SE)*
*Independent variables*										
AF: de jure	0.520	(0.060)***								
AF: de jure (IV: Democracy_t-5_, z)			2.885	(0.402)***						
AF: de jure (IV: Democracy stock_t-10_, z)					2.849	(0.390)***				
AF: de jure (IV: AF_t-5_)							0.628	(0.086)***		
AF: de jure (IV: AF stock_t-10_)									0.611	(0.086)***
*Controls*										
Year/country dummies	Yes		Yes		Yes		Yes		Yes	
Countries (observations)	156	(10,269)	155	(9,883)	154	(9,474)	155	(9,145)	154	(7,941)
R^2^ (R^2^ adjusted)	0.825	(0.820)	0.794	(0.788)	0.798	(0.792)	0.832	(0.827)	0.839	(0.834)

*Notes*: The estimates are obtained from pooled OLS regressions. All variables are described in Table 1. Robust standard errors in parentheses.

* p < 0.10

** p < 0.05

*** p < 0.01.

**Table 13:** The effect of academic freedom (AF) on innovation output (forward citations) with a de jure metric of AF.

Model	(1)		(2)		(3)		(4)		(5)	
Dependent variable	*Forward citations*_*t+5*_ *(ln)*		*Forward citations*_*t+5*_ *(ln)*		*Forward citations*_*t+5*_ *(ln)*		*Forward citations*_*t+5*_ *(ln)*		*Forward citations*_*t+5*_ *(ln)*	
Statistic	*Coeff*.	*(SE)*	*Coeff*.	*(SE)*	*Coeff*.	*(SE)*	*Coeff*.	*(SE)*	*Coeff*.	*(SE)*
*Independent variables*										
AF: de jure	0.280	(0.042)***								
AF: de jure (IV: Democracy_t-5_, z)			6.990	(0.566)***						
vAF: de jure (IV: Democracy stock_t-10_, z)					7.028	(0.538)***				
AF: de jure (IV: AF_t-5_)							0.461	(0.065)***		
AF: de jure (IV: AF stock_t-10_)									0.391	(0.068)***
*Controls*										
Patent applications (ln)	Yes		Yes		Yes		Yes		Yes	
Year/country dummies	Yes		Yes		Yes		Yes		Yes	
Countries (observations)	155	(9,819)	154	(9,433)	153	(9,024)	155	(8,696)	153	(7,500)
R^2^ (R^2^ adjusted)	0.658	(0.648)	-		-		0.679	(0.669)	0.705	(0.695)

*Notes*: The estimates are obtained from pooled OLS regressions. All variables are described in Table 1. Robust standard errors in parentheses.

* p < 0.10

** p < 0.05

*** p < 0.01.

#### 4.4.6 Considering the subdimensions of the academic freedom index

Finally, the academic freedom index is a composite index that is constructed based on country experts’ evaluation of five subdimensions: (1) freedom to research and teach, (2) freedom of academic exchange and dissemination, (3) institutional autonomy, (4) campus integrity, and (5) freedom of academic and cultural expression [46]. To gain more nuanced insights on how these subdimensions relate to innovation output, thereby allowing more nuanced insights into which mechanisms could explain our main results, we perform a final analysis in which we substitute the academic freedom index by these subdimensions.

The results in Table 14 (innovation quantity) and Table 15 (innovation quality) show that all subdimensions are positively associated with innovation output (p < .01). Because all subdimensions are similarly associated with innovation output, it is difficult to draw conclusions about whether some of these subdimensions are more important than others in determining innovation output. Overall, the consistent results, as well as the high correlations between the subdimensions (ranging from 0.75 to 0.95) suggests that it is sensible to combine these subdimensions into a composite academic freedom index, which is our main independent variable.

**Table 14:** The effect of academic freedom (AF) on innovation output (patent applications) when considering the subdimensions of the academic freedom index.

Model	(1)		(2)		(3)		(4)		(5)	
Dependent variable	*Patent applications*_*t+2*_ *(ln)*		*Patent applications*_*t+2*_ *(ln)*		*Patent applications*_*t+2*_ *(ln)*		*Patent applications*_*t+2*_ *(ln)*		*Patent applications*_*t+2*_ *(ln)*	
Independent variables	*Coeff*.	*(SE)*	*Coeff*.	*(SE)*	*Coeff*.	*(SE)*	*Coeff*.	*(SE)*	*Coeff*.	*(SE)*
*Independent variables*										
AF: freedom to research and teach (z)	0.373	(0.030)***								
AF: freedom of academic exchange and dissemination (z)			0.368	(0.032)***						
AF: institutional autonomy (z)					0.298	(0.030)***				
AF: campus integrity (z)							0.364	(0.032)***		
AF: freedom of academic and cultural expression (z)									0.169	(0.025)***
*Controls*										
Year/country dummies	Yes		Yes		Yes		Yes		Yes	
Countries (observations)	157	(12,387)	157	(12,387)	157	(12,448)	157	(12,394)	157	(15,791)
R^2^ (R^2^ adjusted)	0.809	(0.805)	0.811	(0.807)	0.811	(0.807)	0.811	(0.807)	0.791	(0.787)

*Notes*: The estimates are obtained from pooled OLS regressions. All variables are described in Table 1. Robust standard errors in parentheses.

* p < 0.10

** p < 0.05

*** p < 0.01.

**Table 15:** The effect of academic freedom (AF) on innovation output (forward citations) when considering the subdimensions of the academic freedom index.

Model	(1)		(2)		(3)		(4)		(5)	
Dependent variable	*Forward citations*_*t+5*_ *(ln)*		*Forward citations*_*t+5*_ *(ln)*		*Forward citations*_*t+5*_ *(ln)*		*Forward citations*_*t+5*_ *(ln)*		*Forward citations*_*t+5*_ *(ln)*	
Statistic	*Coeff*.	*(SE)*	*Coeff*.	*(SE)*	*Coeff*.	*(SE)*	*Coeff*.	*(SE)*	*Coeff*.	*(SE)*
*Independent variables*										
AF: freedom to research and teach (z)	0.304	(0.020)***								
AF: freedom of academic exchange and dissemination (z)			0.264	(0.021)***						
AF: institutional autonomy (z)					0.201	(0.020)***				
AF: campus integrity (z)							0.263	(0.022)***		
AF: freedom of academic and cultural expression (z)									0.247	(0.015)***
*Controls*										
Patent applications (ln)	Yes		Yes		Yes		Yes		Yes	
Year/country dummies	Yes		Yes		Yes		Yes		Yes	
Countries (observations)	157	(11,916)	157	(11,916)	157	(11,977)	157	(11,923)	157	(15,320)
R^2^ (R^2^ adjusted)	0.643	(0.635)	0.642	(0.633)	0.640	(0.631)	0.642	(0.633)	0.629	(0.623)

*Notes*: The estimates are obtained from pooled OLS regressions. All variables are described in Table 1. Robust standard errors in parentheses.

* p < 0.10

** p < 0.05

*** p < 0.01.

## 5. Conclusion

### 5.1 Discussion

Does academic freedom spur innovation? Although the question has attracted attention in the research-policy literature, the aggregate impact of academic freedom on both the quantity and quality of innovation output around the world is underexplored [37, 40]. Motivated by this research gap, we leverage a novel data source, the academic freedom index, to investigate the relation between academic freedom and innovation in a comprehensive sample of 157 countries over the 1900–2015 period. Employing instrumental variable methods to identify the causal effect of academic freedom on innovation output, we find that academic freedom positively affects the number of patent applications and the number of forward citations. According to our baseline model estimates, improving academic freedom by one standard deviation increases the number of patents two years later by 41% and the number of forward citations five years later by 29%. The results are robust to several sensitivity tests.

To the best of our knowledge, this is the first study that explicitly tests the impact of academic freedom on the quantity and quality of innovation output. Understanding the role of academic freedom in innovation informs the broader policy debates about promoting science [38–40, 55]. Despite the widespread recognition of academic freedom as a fundamental value and principle in academia, concerns have recently emerged about the de facto erosion of academic freedom in higher education and research systems [16, 17, 24]. These worries are, for example, expressed in intensifying public debates about academic freedom and the increasing number of perceived and real violations of academic freedom. Our study provides empirical evidence on the importance of ensuring academic freedom for policymakers and other stakeholders engaged in science diplomacy [37, 56]. Also, this is important for corporate R&D managers as it informs them about a crucial aspect of the geographical location choice of their R&D activities [57].

We present the empirical relation between academic freedom and innovation with the double purpose to stimulate a debate among policymakers and spur future research to deepen our understanding of this stylized fact. For policymakers, our conclusion in combination with the recent decline in academic freedom should be alarming. Academic freedom had progressively increased from the 1940s to the 2010s, but it reversed and started to decline in the last decade both at the global level and in the 25 leading countries in science (see Fig 2). Based on our results, the global decline in academic freedom that occurred in the last decade results in a decreased innovation output, manifesting in both fewer patents applications and patent citations. For researchers, the newly available data on academic freedom across countries paves the way for further investigating the contingency factors of when and how a lack of academic freedom impedes innovation. In this regard, while our exploratory study’s purpose is to establish the academic freedom-innovation relation, future research may explore the various channels through which policymakers may improve academic freedom to promote patenting activity.

### 5.2 Limitations and avenues for future research

To assess the causality of our main results, we use the degree of democracy in a country as an instrument for academic freedom, which is in line with research in political sciences [48–50]. Hence, the exclusion restriction requires that the degree of democracy affects innovation output only through its effect on academic freedom output. However, the relationship between democracy and innovation is the subject of a longstanding debate [58] that is still ongoing. For example, [58: 1272] find that “democracy itself has no direct positive effect on innovation measured with patent counts, patent citations and patent originality”. This supports use of democracy as an instrument. However, this finding is challenged by [51], who do find an association between innovation quantity (they do not assess innovation quality). This suggests a possible violation of the exclusion restriction, at least when it comes to innovation quantity. While we seek to provide an initial comprehensive account of the relationship between academic freedom across countries and years, this ongoing discussion suggests that future research should try to more carefully identify the causal relation between academic freedom and innovation output. For example, future research could explore different instruments or leverage natural experiments to isolate the casual relationship more meticulously. In particular, countries that experienced major political, social, or academic changes that could also have affected academic freedom could serve as promising settings for natural experiments in this regard (e.g., Germany, USSR, or Korea).

Another point that is related to the causal relation between academic freedom and innovation output refers to the mechanisms by which academic freedom affects innovation output. While we mention several mechanisms that could explain the association between academic freedom and innovation output, such as increased creativity and open information exchange, we lack data on empirically test these mechanisms them more thoroughly. This opens various avenues for future research to theorize about and empirically test which mechanisms drive the positive association that we document in this study.

Other concerns relate to the data that we draw on. For example, both our patent data and our academic freedom data cover an extensive period and a substantial number of countries, raising questions about the reliability of the data. While our patent data comes from the commonly used and established database PATSTAT, the number of patent applications and citations recorded could differ in their accuracy in some countries and/or in some years. Similarly, despite the well-documented and robust procedure that was used to generate the academic freedom index, the data is largely based on retrospective ratings by several country experts. This gives rise to potential inconsistencies (e.g., the ratings might not be equally reliable for all countries and/or years). While we mitigate some of these potential biases in or robustness checks, future research should revisit our findings with different indicators to better understand the consequences of changes in academic freedom. Regarding innovation output, other intellectual property rights like trademarks could provide complementary insights [51, 59]. Going beyond the domain of innovation, future research could assess the association between academic freedom and a range of other societally relevant outcome variables, such as entrepreneurship, social change, and sustainability. In the same vein.

Finally, we focus on inventions as one tangible economic consequence of academic freedom, but we do not mean to say that economic considerations are the sole, or even the most relevant implications of academic freedom. Different moral dilemmas and ethical concerns may be triggered by increasing or decreasing academic freedom, respectively. Increasing academic freedom may lead to difficult scenarios of spurring morally debatable technologies (e.g., warfare or cloning technologies), while decreasing academic freedom may lead to a relative shortage of inventions, potentially to the detriment of society from a utilitarian perspective. Thus, an ethical debate of the interplay between academic freedom and innovation in universities and corporations seems to be a very promising way to advance this literature.
